# Treatment of Granular Ophthalmia, by the Liberal Use of Quinine

**Published:** 1872-04

**Authors:** C. Bader

**Affiliations:** Ophthalmic Assistant Surgeon, Guy’s Hospital


### TREATMENT OF GRANULAR OPHTHALMIA BY THE LOCAL USE OF QUININE, (QUINIÆ BISULPHAS).

BY C. BADER, OPHTHALMIC ASSISTANT SURGEON, GUY'S HOSPITAL.

The unsatisfactory results of the treatment of granular ophthalmia, especially if combined with pannus, (vascular cornea), will, Mr. Bader hopes, justify the publication of the following remarks. Experiments were made with various substances on patients suffering from granular ophthalmia with pannus. The idea which led to the selection of the substances experimented with was, that granular ophthalmia is the result of some extraneous substance becoming lodged in the conjunctiva, and giving rise to what have been called granulations. Experiments made elsewhere with antiseptics on organic matter have shown quinine to be one of the most effective antiseptics.

The substances experimented with on granular ophthalmia were the scrapings and juice of the root of bryonia nigra, the nitric oxide of mercury ointment (hydrarg. nitrico-oxidi, gr. iij, adipis dr. j), and the bisulphate of quinine in powder. The remedies were applied morning and evening; of the quinine about as much as would go on the point of a penknife was placed, with a dry camel-hair brush, on the inner surface of each lower eye-lid. No other treatment, such as exclusion from light, lotions, etc., was made use of. The hypothesis that quinine acts specifically upon granulations, as mercury, for instance, does on lice, may be erroneous; the effect may merely be that of an irritant, causing moderate suppuration, and with it, removal of the granulations.

The effect on granular conjunctiva of bryonia nigra and of the nitric oxide of mercury ointment, though curious in other respects, are of little interest here. Suffice it to state those of quinine. In some cases its application was followed by severe smarting, which continued for ten or fifteen minutes; in other cases no pain whatever was felt; in all cases appeared increased purulent discharge from the conjunctiva, with shrinking of the granulations and clearing of the surface of the cornea. The intolerance of light ceased rapidly in all cases; the dilatation of the pupils appeared in from twelve to twenty-four hours after the first application of quinine. The pupils, though dilated in ordinary light, contracted well on exposure to strong light.

Case 1.—A. B. aged fourteen. Granular ophthalmia for six years; at present vascular cornea (pannus), in both eyes; pupils barely visible; granular conjunctiva; extreme intolerance of light. The application of bryonia nigra was followed by symptoms of purulent ophthalmia (chemosis, swollen lids, etc.), which, with slight improvement of sight, subsided within ten days; the intolerance of light persisted. Quinine was used and the bryonia discontinued. The eyes improved rapidly; the granulations became smaller, and the intolerance of light disappeared completely within four days.

Case 2.—C. D., aged ten. Granular ophthalmia with pannus, for two years, with intolerance of light. The use of the nitric oxide of mercury ointment (for fourteen days) was followed by slight improvement. It was discontinued and quinine substituted. Within ten days all intolerance of light had ceased, and the cornea became much clearer, etc.

Case 3.—E. F., ten years. Granular ophthalmia for one year. The intolerance of light persisted for three weeks, while the nitric oxide of mercury ointment was used; it disappeared on the second day after the quinine had been applied.—*Lancet.*

